# Perceived influence of alcohol consumption, substance use, and mental health on PrEP adherence and condom use among PrEP-prescribed gay, bisexual, and other men-who-have-sex-with-men: a qualitative investigation

**DOI:** 10.1186/s12889-022-14279-2

**Published:** 2022-10-07

**Authors:** Paul A. Shuper, Thepikaa Varatharajan, David J. Kinitz, Dionne Gesink, Narges Joharchi, Isaac I. Bogoch, Mona Loutfy, Jürgen Rehm

**Affiliations:** 1 Centre for Addiction and Mental Health (CAMH), Institute for Mental Health Policy Research & Campbell Family Mental Health Research Institute, 33 Russell St., Toronto, ON M5S 2S1 Canada; 2 Dalla Lana School of Public Health, University of Toronto, 155 College St., Toronto, ON M5T 3M7 Canada; 3 School of Public Health Sciences, University of Waterloo, Waterloo, ON Canada; 4 Divisions of General Internal Medicine and Infectious Diseases, University Health Network, 200 Elizabeth St., Toronto, ON M5G 2C4 Canada; 5 Department of Medicine, Medical Sciences Building, University of Toronto, 1 King’s College Circle, Toronto, ON M5S 1A8 Canada; 6 Women’s College Hospital, 76 Grenville S.t, Toronto, ON M5S 1B2 Canada; 7 Maple Leaf Medical Clinic, 14 College St., Toronto, ON M5G 1K2 Canada; 8 Department of Psychiatry, University of Toronto, 250 College St., Toronto, ON M5T 1R8 Canada; 9 Center for Interdisciplinary Addiction Research (ZIS), Department of Psychiatry and Psychotherapy, University Medical Center Hamburg-Eppendorf (UKE), Martinistraße 52, 20246 Hamburg, Germany; 10 Institute of Clinical Psychology and Psychotherapy, Technische Universität Dresden, Chemnitzer Straße 46, 01187 Dresden, Germany; 11 Faculty of Medicine, Institute of Medical Science, Medical Sciences Building, University of Toronto, King’s College Circle, Room 2374, Toronto, ON M5S 1A8 Canada; 12 Program On Substance Abuse & WHO CC, Public Health Agency of Catalonia, 81-95 Roc Boronat St., 08005 Barcelona, Spain; 13 I.M. Sechenov First Moscow State Medical University (Sechenov University), Trubetskaya Street 8, b. 2, Moscow, 119991 Russian Federation

**Keywords:** HIV Pre-Exposure Prophylaxis (PrEP), Adherence, Alcohol, Substance Use, Mental Health, Gay/Bisexual/MSM

## Abstract

**Background:**

Gay, bisexual, and other men-who-have-sex-with-men (GBMSM) continue to be disproportionately affected by Human Immunodeficiency Virus (HIV). Although HIV pre-exposure prophylaxis (PrEP) offers an effective means of reducing incident HIV among this population, the HIV-preventive success of oral-based PrEP is contingent upon regimen adherence. Elevated rates of alcohol-, substance use-, and mental health-related issues among GBMSM potentially hinder PrEP-taking efforts, however the evidence for this remains mixed. Accordingly, the present study entailed a comprehensive qualitative investigation to explore PrEP-prescribed GBMSM’s perceptions surrounding the influence of alcohol, substance use, and mental health on PrEP adherence.

**Methods:**

PrEP-prescribed GBMSM (age ≥ 18 years; prescribed PrEP for ≥ 3 months) were recruited from two PrEP-delivery clinics in Toronto, Canada for focus groups as part of the formative phase of an alcohol-, substance use-, and mental health-focused randomized controlled intervention trial. Focus group discussions qualitatively explored perceived strengths and barriers associated with adherence to PrEP treatment; with an emphasis on alcohol, substance use, and mental health concerns. Condom use among PrEP-prescribed GBMSM within the context of these concerns was also discussed.

**Results:**

A total of five focus groups involving 35 GBMSM were conducted (4–10/group; mean age = 42.4; white = 71.4%). Although participants themselves generally reported successfully adhering to their PrEP regimens—resulting from a strong, underlying motivation for self-care—they recognized the detrimental impact that alcohol, substance use, and mental health had on adherence among their peers. In this regard, alcohol and substances were perceived as detracting from adherence only when consumption was excessive or temporally linked to PrEP dosing. Pronounced mental health issues (e.g., severe depression) were also seen as hindering adherence, although these effects were nuanced and perceived as person-dependent. Alcohol and substances were linked to condomless sex, regardless of PrEP use, and PrEP was therefore viewed as an HIV-protective ‘safety net.’

**Conclusions:**

Overall, findings suggest that PrEP adherence can often be successfully achieved in the presence of alcohol-, substance use-, and mental health-related issues. Augmenting self-care, and addressing pronounced addictions- and mental health-related concerns, may enhance PrEP treatment among GBMSM.

## Background

Despite a modest decline in the global incidence of human immunodeficiency virus (HIV) in recent years [1], key populations continue to be disproportionately affected by the virus. In particular, incident HIV remains pronounced among populations of gay, bisexual, and other men-who-have-sex-with-men (GBMSM), who comprised 23% of new HIV infections globally in 2019 [2]. This disparity is especially evident in countries such as the United States and Canada, where recent surveillance data have demonstrated that GBMSM comprise 69% [3] and 41% [4] of all new HIV infections, respectively.

Within this context, HIV pre-exposure prophylaxis (PrEP) serves as a crucial component that can considerably help curtail incident HIV among GBMSM. PrEP typically entails oral dosing of tenofovir disoproxil fumarate and emtricitabine (TDF/FTC) or tenofovir alafenamide and emtricitabine (TAF/FTC), which can markedly reduce the likelihood of acquiring HIV if exposed to the virus [5–11]. PrEP’s ability to prevent HIV acquisition, however, has been shown to be strongly associated with regimen adherence, for both daily [6, 8, 9, 12–15] and non-daily PrEP regimens [8, 16]. Although a long-acting injectable form of PrEP has recently emerged that eliminates the requirement for daily oral dosing [17], its regulatory approval currently remains limited to a small number of jurisdictions. As a result, sustained adherence to oral dosing of TDF/FTC or TAF/FTC remains a necessity for the broader population of PrEP users.

Behavioral and psychosocial barriers may hinder PrEP-taking efforts [18, 19]; with the consumption of alcohol and/or substances, as well as the experience of mental health concerns such as depression, potentially serving as considerable challenges. The prevalence of these barriers tends to be higher among GBMSM, including GBMSM who have been prescribed PrEP, compared to the general population [20–25]; which in part may derive from a range of unique stressors and socio-contextual factors that GBMSM experience (e.g., [26]). However, while alcohol, substance use, and mental health issues have all been significantly associated with poorer adherence to antiretroviral therapy (ART) among GBMSM who are living with HIV [27, 28], the associations between these issues and adherence specifically to PrEP have been somewhat less consistent.

On the one hand, evidence from some quantitative and qualitative investigations has provided support for associations between lower PrEP adherence and alcohol consumption [29–34], substance use [33–41], and depression [40–43]; suggesting that these factors may hinder one’s motivation and/or ability to take PrEP as prescribed. In contrast, findings from other studies have demonstrated that some GBMSM are able to successfully adhere to their PrEP regimens, even when experiencing addictions- and mental health-related concerns [36–39, 44, 45]. Within this regard, it is possible that these latter individuals may have adopted unique strategies (e.g., taking PrEP before using a substance, taking PrEP as part of a pre-sex routine) that enable them to maintain PrEP adherence in spite of underlying challenges [34, 35, 44]. Alternatively, it may be the case that the degree of severity with which these addictions- and mental health-related challenges are manifested influences the extent of the associations with PrEP-taking behaviors [33, 46]. A third possibility is that individuals who consume alcohol or substances may be cognizant of their increased likelihood of engaging in condomless sex, and in turn amplify their PrEP-taking efforts [45].

Interestingly, this latter supposition highlights the added complexity surrounding condom use decisions among PrEP-prescribed GBMSM who may be experiencing issues involving alcohol, substances, and/or mental health. Despite PrEP’s ability to serve as an additional means of HIV protection among such individuals [47], the presence of addictions- and mental health-related issues could potentially inhibit both PrEP adherence and the use of condoms, resulting in an elevated risk of HIV acquisition. The diminished use of condoms can also elevate one’s risk of acquiring other sexually transmitted infections (STIs), including chlamydia, gonorrhea, and syphilis.

Taken together, greater clarity is needed to delineate the complex interplay of alcohol, substance use, and mental health in relation to PrEP adherence and condom use among PrEP-prescribed GBMSM. The present study entailed a comprehensive, qualitative investigation to explore the dynamics that underpin this interplay.

## Methods

### Data collection

As part of the formative research phase of a randomized-controlled intervention trial (ClinicalTrials.gov Identifier: NCT05097430), men from two clinics providing PrEP in Toronto, Canada were recruited through convenience sampling to participate in one of five focus group discussions on PrEP-related experiences. The recruitment process involved clinic staff mentioning the study to their patients and referring those were interested to a study research team member, who then provided detailed information about the study and arranged participation in a focus group session. Eligibility criteria included 1) age ≥ 18 years; 2) identifying as GBMSM; and 3) prescribed PrEP for ≥ 3 months.

Participants provided informed written consent and completed a brief demographic survey for sample characterization (e.g., age, race/ethnicity, education). Semi-structured focus group discussions (~ 2 h in length) were held in person to explore strengths and barriers impacting participants’ adherence to daily PrEP treatment and use of condoms; with an additional emphasis on alcohol, substance use, and mental health. Focus groups were conducted by a Professor in Epidemiology who works in the area of sexual health and possesses extensive experience conducting focus groups with diverse populations, including GBMSM and other marginalized groups. All sessions were audio-recorded, and recordings were transcribed verbatim and reviewed for accuracy. Participants received CAD $50 (~ USD $40) for taking part. Procedures were approved by Research Ethics Boards at the Centre for Addiction and Mental Health (#101/2017) and the University Health Network (#18–5014).

### Data analysis

Summary statistics of survey responses were generated through SPSS [48] to describe the study sample. A combined deductive/inductive thematic analysis was employed to analyze focus group transcripts with the intention of developing an in-depth understanding about the roles of alcohol, substance use, and mental health in relation to daily PrEP adherence and condom use ([49], p. 86). A deductive approach was taken to explore themes identified from the research question and topics addressed by the focus group guide, while an inductive analysis was also implemented to identify new themes interpreted from the data. The analytic process included data familiarization, developing a coding framework based on established and newly identified topics, iterative development of themes, a review of themes by the research team, finalization of themes, and writing [49].

NVivo was used to store, code, and organize focus group data [50]. A preliminary list of broad categories was developed based on the research question a priori by the study’s Principal Investigator (PI), Co-Investigators, and Research Coordinator, who possess expertise and experience in the fields of HIV, mental health, and addictions. Two graduate-level Research Assistants trained in qualitative analysis actively employed several analytic strategies, including deeply familiarizing with the transcripts, conceptualizing the data in relation to scholarship, theory, and accounts within and across the transcripts, and taking a team approach [51]. A subgroup of the research team, which included the PI, Research Coordinator, and Focus Group Facilitator, was consulted after the Research Assistants had coded the first two focus groups to discuss the identified codes, possible themes, and resolve discrepancies between coders [52]. A codebook was developed once all focus groups had been analyzed. Codes were collated to generate categories and themes across the data set, and themes were reviewed and refined by the above-mentioned research team subgroup and Research Assistants. Rich descriptions and illustrative quotes were used to convey participants’ experiences and opinions and researchers’ interpretations.

## Results

Five focus groups with 35 PrEP-prescribed GBMSM (4–10 participants/group) were conducted from June to August 2018. Socio-demographic characteristics can be found in Table 1. Participants had a mean age of 42.4 years, 71.4% were white, and average household income was CAD $112,700 (~ USD $90,160). Roughly three quarters of participants (76.5%) had been using PrEP for more than 12 months.

**Table 1:** Sample Demographics (*n* = 35)

Characteristic	n (%)^a^
Age	
≤ 29	3 (8.6%)
30–39	15 (42.9%)
40–49	5 (14.3%)
≥ 50	12 (34.3%)
Race/Ethnicity	
White	25 (71.4%)
Chinese	2 (5.7%)
South Asian (e.g., East Indian, Pakistani, Sri-Lankan, etc.)	1 (2.9%)
Black	2 (5.7%)
Filipino	1 (2.9%)
Latin American	3 (8.6%)
Multi-race	1 (2.9%)
Sexual Orientation	
Gay	33 (94.3%)
Bisexual	1 (2.9%)
Pansexual	1 (2.9%)
Currently Have a Steady Partner	11 (31.4%)
Annual Household Income (CAD)	
≤ $59,999	6 (17.1%)
$60,000—$79,999	7 (20.0%)
$80,000—$99,999	6 (17.1%)
≥ $100,000	16 (45.7%)
Highest Level of Education	
Completed high school (received secondary school diploma)	1 (2.9%)
Some trade or technical training	1 (2.9%)
Completed trade or technical training (received certification/diploma)	2 (5.7%)
Some college	1 (2.9%)
Completed college (received degree or diploma)	3 (8.6%)
Some university	2 (5.7%)
Completed university (received degree)	18 (51.4%)
Post-graduate education	7 (20.0%)
Duration on PrEP (months)	
< 12	8 (23.5%)
12—< 24	10 (29.4%)
24—< 36	10 (29.4%)
≥ 36	6 (17.6%)

^a^ Percentages are based on the number of participants who indicated a specific response divided by the number of participants who responded to the question. CAD: Canadian dollar (CAD $1.00 =  ~ USD $0.80)

### PrEP adherence

Adherence was discussed in terms of daily dosing, as focus group participants had been prescribed daily PrEP regimens. Within this context, although using alcohol and substances and experiencing poorer mental health were seen as having the potential to impact PrEP adherence in the general PrEP-prescribed GBMSM population, study participants themselves did not appear to let challenges detract them from their PrEP use. Participants reported being concertedly dedicated to their health and wellbeing; prioritizing self-care (i.e., maintaining adherence) regardless of encountered barriers such as “bout(s) of depression” (Focus Group 1 (FG1)) or casual drinking/substance use. One participant described the relationship between his mental health and PrEP adherence:

*“When I’ve had sort of different varying mental health stages, I’ve been able to stay on pills but that’s because I don’t see it as something that is hard to do when I’m maybe going through a challenging bout of whatever.”* (FG3)

Another participant shared a similar sentiment about the relationship between his alcohol and substance use and PrEP adherence:

*“I smoke weed every day and I’m a social drinker. Never would I think, “Oh, too drugged to open a pill box and make sure it goes down and keep it down.”* (FG4)

However*,* when speaking of peers’ experiences with poor mental health or using alcohol or substances, participants identified the potential for these aspects to have an impact on their ability to maintain PrEP use. For example, one participant said, *“If you’re drunk a lot, you’re susceptible to forgetting to take your pill.”* (FG5) Another participant shared:

*“I have a number of friends who have been through episodes of mental illness and continued mental illness…I would say it definitely would impact because you become so absorbed in your own cyclone of personal issues that I think things could go by the way…including taking medications.”* (FG2)

Two themes related to alcohol, substance use, and PrEP adherence were identified – the degree of intoxication, and the temporal overlap of alcohol/substance use and PrEP dosing. Additionally, two themes related to mental health and PrEP adherence were described—severity of mental health, and the motivation to adhere when experiencing mental health issues.

#### Degree of alcohol- and substance-related intoxication

Men noted that the ability to adhere to PrEP depends on one’s level of intoxication. Participants felt that men who are drunk or high are probably not going to be able to perform the necessary *“checks and balances”* (FG5) that allow them to remember to take their pill. One participant described:

*“If you’re supposed to take a pill once a day or whatever time of day but you’ve gone out and you’re like totally hammered and drunk and omg you come home and you just pass out. Then you might not take your pill obviously…I think there’s different levels of being intoxicated.”* (FG3)

Participants also reported that taking PrEP on a daily basis can be a barrier for men who have marked substance use issues compared to those who use occasionally. Participants felt that men who are dependent on alcohol or substances may be engaging in self-destructive behaviors that prevent them from focusing on their health and keeping up with their PrEP care. One participant stated:

*“I guess the only real barrier is somebody who actually has an addiction problem. That’s a different case. I don’t think if somebody’s drinking on the weekend then that’s stopping them from going on PrEP in any way.”* (FG1)

#### Temporal overlap of alcohol/substance use and PrEP dosing

A barrier to adherence noted by participants was the overlap between the timing of PrEP doses and the consumption of alcohol or substances. Participants suggested that men who are out partying and using substances may not think about taking their pill on schedule. For example:

*“You’re high at the time that you should be taking your pill and when your high is gone it’s the next day and you’ve actually missed an entire dose and you’re not going to have the foresight to do it ahead of time and you’re not going to think back and be like, “oh well I didn’t take a pill.” You’re just going to wait for the next time to take your pill. So you’re in effect, skipping doses.”* (FG3)

However, participants also offered solutions, such as changing the timing of PrEP dosing so that there was no conflict. As one participant shared, *“I don’t know—if a friend came to me and was like, “I always miss my doses.” Like, well are you getting high at 9am? And if you are, great, take the dose at 8.”* (FG3).

#### Severity of mental health

The severity of mental health issues was identified as a theme associated with PrEP adherence. Some study participants reported diagnosed mental illnesses (e.g., depression, bipolar disorder) or poorer mental health in general and also reported few issues with adherence themselves. However, these same participants also noted that mental health may have a detrimental impact on PrEP adherence among individuals experiencing a severe mental health episode. As one participant described, “*Depending on what they’re going through with their mental health…taking a pill might be too big of a deal.*” (FG3).

#### Motivation to adhere when experiencing mental health issues

Participants described how experiencing poor mental health (e.g., depression) could lead to a lower sex drive and fewer sexual encounters, and how during these periods, one might question why they should continue taking PrEP. For instance, one participant shared,*“…for me, if I’m not feeling good or whatever…I’m probably not having sex. In that case, I would think why should I bother? Why should I even continue taking this?”* (FG2).

Meanwhile, others who felt their sexual behavior was impacted by their mental health did not believe that their daily routines, including taking PrEP, were compromised. An overall commitment to ongoing self-care was consistent through sentiments of: *“…well, I’m just going to take this.”* (FG3). One participant explained:

*“I mean I didn’t have as much of a high sex drive anyways during that period of time but I still saw the need to take my PrEP even though I wasn’t even having sex at the time. Just because that was just a part of my lifestyle at that time.”* (FG4)

### Condom use

Two themes emerged around the relationship between the consumption of alcohol and substances and the use of condoms: a generalized increase in condomless sex when intoxicated, regardless of taking PrEP; and relying on PrEP as a means of protection when intoxicated. Similarly, two mental health-related themes emerged: individualized perceptions regarding the links between mental health and condom use; and PrEP’s protective benefits among those experiencing mental health issues.

#### Increased condomless sex while intoxicated, regardless of PrEP use

Some participants reported that their alcohol and substance use had more of an impact on their ability to use condoms than to adhere to PrEP. Participants reported that irrespective of being on PrEP, using alcohol or substances impacted both the ability to evaluate risk and the resultant engagement in condomless sex. As one participant explained, *“If you’re drunk and high and you’re even that much more inebriated and not able to make a proper responsible decision.”* (FG1) This sentiment was personalized by another participant who shared, *“Personally, if I’m a little drunk or high or whatever, I will be less likely to use a condom.”* (FG2).

#### PrEP as a means of protection while intoxicated

Participants felt that taking PrEP was their way of balancing being responsible with feeling free by preemptively preparing for risky situations that involved lowered inhibitions and condomless sexual encounters. As one participant described,

*“Some of them will start to take PrEP because they want to feel a little bit more protected and exactly feel freer to just keep drinking or taking more drugs and having sex or looking for sex as a consequence of that.”* (FG1).

Another participant disclosed that he started to take PrEP after his sexual encounter with a man who was HIV-positive, which transpired because of his alcohol use.

#### Mental health, condom use, and PrEP – a range of perceptions

Interestingly, unlike alcohol and substance use, mental health was not consistently associated with condomless sex among PrEP users. Instead, responses were highly individualized; reflecting diverse perceptions ranging from inconsequential to important. One participant shared:

*“I’ve never felt that my depression or the state of my mental health has had an impact on the sexual choices I make and my behaviors.”* (FG1)

Another shared:

*“Whether or not condom use happens depends upon their own sexual behaviors and whatever they might be dealing with.”* (FG1)

#### PrEP as an additional layer of protection when experiencing mental health issues

Participants shared that they take PrEP as a ‘safety net’ (FG3) for times where they are in a state of depression or apathy and consequently are not as concerned about potentially riskier sexual practices. One participant described,

*“…if you’re already at a point where your sort of level of self-care isn’t great or you’re depressed or you’re suicidal…it’s sort of…something like, ‘oh well. So, what? So, what if this happens?’...so PrEP for me has sort of been this sort of ‘safety net.’”* (FG3)

## Discussion

Findings suggest that study participants tended to be highly motivated to maintain their PrEP care, and that PrEP adherence can be successfully achieved for many GBMSM who consume alcohol, use substances, or experience depression. However, participants speculated that consistent PrEP use may be challenging for those whose alcohol consumption or substance use is excessive or temporally linked to PrEP dosing, or whose mental health has deteriorated to the point that they are no longer taking care of themselves and the motivation to maintain their health and well-being is diminished. The use of alcohol and substances was also recognized as a barrier to using condoms during sex, but this detrimental impact was perceived to be present regardless of being on PrEP. Within this regard, taking PrEP was deemed as an effective ‘safety net’ to prevent HIV acquisition.

Our findings support the use of PrEP for HIV prevention among GBMSM, despite this population’s disproportionate burden of addictions and mental health concerns [20–25, 53, 54]. Results not only accord with previous research (e.g., [45]) by demonstrating that concerted adherence efforts may be undertaken to counteract the greater likelihood of condomless sex while under the influence or when experiencing mental health issues, but they also extend this work and provide new insight by suggesting that the possession of a strong, underlying motivation for “self-care,” reflecting one’s attitudes and behaviors relevant for the prevention or self-management of a health-related concern [55], can help surmount the challenges that alcohol, substance use, and mental health issues potentially pose on PrEP-taking efforts. Self-care in general [55], along with associated constructs involving one’s motivation to stay healthy [56] and one’s focus on health-promotion and healthy practices [57], have been identified as key facilitators of HIV treatment and HIV-related resilience. Furthermore, interventions that promote a range of self-care-focused behaviors have been shown to be associated with improvements in ART adherence among people living with HIV (PLWH) [58]. Addressing precursors of diminished self-care, including, for example, poor coping skills, social support-seeking behaviors, and stigma management [59, 60], may also be beneficial. Accordingly, within the context of PrEP, offering self-care-associated interventions to GBMSM who have difficulty following their regimen, particularly those who also report concerns surrounding alcohol, substance use, or depression, could prove to be an effective means of maintaining long-term adherence among this group.

Importantly, however, while such self-care-promotive interventions may be beneficial for GBMSM who experience relatively less-pronounced alcohol-, substance use-, and mental health-related issues, findings from both the present study and previous research [33, 46] suggest that adherence will likely remain challenging for those with relatively more severe issues. As such, the impact of self-care interventions on their own may be insufficient to improve adherence among this latter group. Therefore, for these individuals, a potentially effective approach could follow methods successfully employed with PLWH, in which the delivery of addictions- and mental health-focused interventions results in the amelioration of the associated underlying conditions, which in turn leads to corresponding improvements in ART adherence [61, 62]. Alternatively, for PrEP-prescribed GBMSM who do not want to reduce their alcohol or substance use, a second approach could entail offering interventions that enhance behavioral skills for taking PrEP in the context of ongoing consumption. For example, as suggested by the present results, adherence could be enhanced by first recognizing one’s alcohol and substance use patterns, and then arranging one’s dosing schedule accordingly (see also [35]).

The nuanced relationship described between depression and adherence aligns with disparate findings from previous research [33, 42, 43, 46]. While it is clear that some PrEP-prescribed GBMSM can follow their regimens even when feeling depressed as a result of their strong motivation to maintain self-care, for others, depression may substantially diminish this motivation; leading not only to missed doses, but also to a reduction in condom use when engaged in sexual activity [63]. Despite a reduced desire to have sex when depressed, the potential HIV-related impact of depression remains concerning, given that for some GBMSM, depression-induced diminished self-care may lead to sexual situations that are neither PrEP- nor condom-protected. As such, additional research remains necessary to identify the spectrum of mechanisms that underpin the association between depression and PrEP adherence [43], which in turn could help target depression-focused as well as adherence-promotive intervention efforts to those PrEP-prescribed GBMSM who may benefit from them the most.

Results should be viewed in light of possible limitations. First, recruitment was based on convenience sampling, and participation required travelling to a separate hospital site to take part in a session and then openly discussing one’s PrEP-related experiences. Participants therefore required sufficient ability and motivation to attend a session and disclose potentially sensitive information. As a result, our sample may not have reflected the diversity of GBMSM who were receiving PrEP care at our two clinic sites, or the broader population of PrEP-prescribed GBMSM. Second, the sample was also predominantly white, educated, and high-income, which may have further impacted perspectives on and experiences with PrEP adherence, and in turn, the transferability of the findings. Third, to learn about the experiences of a broad range of PrEP users, recruitment was not limited to those who experienced challenges involving PrEP adherence, alcohol consumption, substance use, or mental health. Those who experience such challenges may also struggle to plan and keep an appointment to participate in a research study. This likely impacted the responses yielded through our focus groups, in which participants typically did not report experiencing pronounced issues pertaining to PrEP adherence, alcohol use, or substance use, but acknowledged that such issues existed among some of their friends and peers who were taking PrEP. Restricting participation to those for whom such issues were considerable may have provided additional unique insights. Fourth, when discussing mental health, participants primarily spoke about depression or feeling mentally unwell, and did not mention mental illnesses such as personality disorders or psychosis. The impact of these latter, and in some cases, far more severe forms of mental illness on PrEP treatment requires further exploration. Finally, during focus groups, the concept of adherence was discussed in terms of taking one’s daily PrEP dose, as participants had been prescribed daily regimens. The additional inclusion of participants who had been prescribed non-daily PrEP regimens may have provided unique insight regarding the impact of alcohol, substance use, and mental health on taking PrEP only in conjunction with the engagement in sexual activity (i.e., 2–1-1 regimen) or when following other intermittent PrEP dosing schedules. Furthermore, expanding the concept of PrEP adherence to “prevention-effective adherence,” [64] which accounts for varying periods of risk exposure and the use of other prevention methods (e.g., treatment-as-prevention), may also have provided further insight.

## Conclusions

Findings from the present investigation support the notion that adherence to PrEP can be successfully achieved in the presence of alcohol consumption, substance use, and mental health issues, and may even be ideally suited for such contexts given the ability to use PrEP as an HIV-protective ‘safety net’ that can counteract the corresponding increased likelihood of condomless sex when intoxicated or mentally unwell. Interventions that promote self-care among PrEP-prescribed GBMSM, as well as interventions that address pronounced alcohol, substance use, and mental health concerns, could enhance PrEP treatment efforts among this population.

## Data Availability

The datasets generated during and/or analyzed during the current study are not publicly available due to research ethics-related requirements but may be available from the corresponding author on reasonable request.

## Abbreviations

ART: Antiretroviral therapy
GBMSM: Gay, bisexual, and other men-who-have-sex-with-men
HIV: Human Immunodeficiency Virus
PLWH: People living with HIV
PrEP: Pre-Exposure Prophylaxis
STI: Sexually transmitted infection
TAF/FTC: Tenofovir alafenamide/emtricitabine
TDF/FTC: Tenofovir disoproxil fumarate/emtricitabine
